# Do Questions Reflecting Indoor Air Pollutant Exposure from a Questionnaire Predict Direct Measure of Exposure in Owner-Occupied Houses?

**DOI:** 10.3390/ijerph7083270

**Published:** 2010-08-23

**Authors:** C.K. Jennifer Loo, Richard G. Foty, Amanda J. Wheeler, J. David Miller, Greg Evans, David M. Stieb, Sharon D. Dell

**Affiliations:** 1 University of Toronto, 1 King’s College Circle, Toronto, Ontario M5S 1A8, Canada; E-Mail: jennifer.loo@utoronto.ca (C.K.J.L.); 2 The Hospital for Sick Children, 555 University Avenue, Toronto, Ontario M5G 1X8, Canada; E-Mail: richard.foty@sickkids.ca (R.G.F.); 3 Health Canada, 269 Laurier Ave West, Ottawa, Ontario K1A 0K9, Canada; E-Mails: amanda.wheeler@hc-sc.gc.ca (A.J.W.); Dave_Stieb@hc-sc.gc.ca (D.M.S.); 4 Chemistry Department, Carleton University, Ottawa, Ontario K1S 5B6, Canada; E-Mail: david_miller@carleton.ca (J.D.M.); 5 University of Toronto, 200 College Street, Toronto, Ontario M5S 2E5, Canada; E-Mail: greg.evans@utoronto.ca (G.E.)

**Keywords:** allergens, environmental exposure, house dust, indoor air pollution, questionnaire

## Abstract

Home characteristic questions are used in epidemiological studies and clinical settings to assess potentially harmful exposures in the home. The objective of this study was to determine whether questionnaire-reported home characteristics can predict directly measured pollutants. Sixty home inspections were conducted on a subsample of the 2006 population-based Toronto Child Health Evaluation Questionnaire. Indoor/outdoor air and settled dust samples were analyzed. Mean Fel d 1 was higher (p < 0.0001) in homes with a cat (450.58 μg/g) *versus* without (22.28 μg/g). Mean indoor NO_2_ was higher (p = 0.003) in homes with gas stoves (14.98 ppb) *versus* without (8.31 ppb). Self-reported musty odours predicted higher glucan levels (10554.37 μg/g *versus* 6308.58 μg/g, p = 0.0077). Der f 1 was predicted by the home’s age, but not by reports of carpets, and was higher in homes with mean relative humidity > 50% (61.30 μg/g, *versus* 6.24 μg/g, p = 0.002). Self-reported presence of a cat, a gas stove, musty odours, mice, and the home’s age and indoor relative humidity over 50% predicted measured indoor levels of cat allergens, NO_2_, fungal glucan, mouse allergens and dust mite allergens, respectively. These results are helpful for understanding the significance of indoor exposures ascertained by self-reporting in large epidemiological studies and also in the clinical setting.

## 1. Introduction

Asthma is the most common chronic childhood disease in North America; its prevalence is increasing, and it is a leading cause of emergency department visits, hospitalizations and school absenteeism [1–3]. Because North American children spend a significant proportion of their time indoors at home [4], the role of the home environment in the triggering and exacerbation of childhood asthma has been studied extensively. For example, carpeted floors in homes tend to harbour greater levels of dust mites, which are known to be a risk factor for asthma [5,6]. High dust weight alone has also been associated with increased respiratory symptoms [7]. Dampness and mould exposure in the home [8,9], cat exposure [10], exposure to cockroach allergens [11,12], indoor particulate matter exposure [13], as well as the presence of gas stoves and elevated nitrogen dioxide levels [14,15] are all known to exacerbate childhood asthma. High endotoxin exposures in the home have been associated with increased asthma severity [16,17] and recent research suggests that endotoxins may also be a risk factor for the development of childhood asthma [18–20]. Exposure to mouse allergens in the home has also recently been reported to be associated with asthma morbidity and an increased risk of wheeze in childhood [21–23]. Consequently, environmental modification forms a major part of present asthma management guidelines [24,25].

In the process of gathering indoor air pollutant exposure data, both home inspections and questionnaires can be used. Home inspections assess the direct presence of specific pollutants and may involve dust sampling and air pollution monitoring. Questionnaires, on the other hand, focus on housing characteristics that tend to be associated with pollutant exposures. To ensure that responses serve as adequate predictors of actual exposure, questionnaires must be validated. A review of the literature reveals that questionnaire reports of specific aspects of the home environment, such as a cat in the home, cockroaches, dampness and mould, and the presence of a gas range have been associated with their intended measures of exposure of increased cat allergens, cockroach allergens, fungal concentrations, and nitrogen dioxide, respectively [26–29]. However, there have also been conflicting reports in the literature with regards to whether certain self-reported housing characteristics predict actual measurements. For example, studies conducted in different countries have reported inconsistent results with regards to the ability of the number and type of pets to predict endotoxin levels [30,31]. There have also been differing reports on whether certain housing characteristics, such as humidity, predict dust mite levels [32]. Some of these discrepancies may be due to differences in geographic region or in the population studied (e.g., a low-income population *versus* the general population). It is therefore important to carry out direct measurements of pollutants in samples of subjects that are representative of study populations. This would assist the interpretation of home environment questions in a particular study setting and population. In Canada, where 79% of children reside in urban settings [33], it is vital to determine what air pollutants are present in homes at what levels, how they compare to other countries and settings, and the validity of self-reporting pertaining to these pollutants assessed via questionnaire.

The purpose of this study is to validate questions that reflect indoor air pollutant exposure in the Toronto Child Health Evaluation Questionnaire (T-CHEQ). Questionnaire responses are compared to data obtained through direct measurements during home inspections. Specifically, this research seeks to determine whether certain characteristics indicated on the questionnaire (presence of a gas stove, cat, any other pets, carpets, air conditioner, cockroaches, mice, damp spots, visible moulds or fungi) predict, respectively, increased levels of NO_2_, cat allergen, endotoxin, dust mite allergens, cockroach allergens, mouse allergens, and fungi.

## 2. Methods

### 2.1. Study Population

The Toronto Child Health Evaluation Questionnaire (T-CHEQ) is a population-based study that examines the relationship between air pollution and childhood asthma [34]. Between January and May of 2006, questionnaires were completed by parents of 5,559 grade 1 and 2 schoolchildren to assess asthma prevalence. A section of the T-CHEQ asked parents to answer questions reflecting indoor air pollutant exposures. Some of this data was used to predict indoor concentrations of air pollutants. During August 1 to November 24, 2006 and July 4 to 18, 2007, home inspections were conducted on a subsample of 60 homes that were randomly selected from the T-CHEQ population. This subsample included only families who did not live in apartments, who did not smoke inside the home, and who owned their homes. Smokers were excluded from the subsample because cigarette smoke has already been well-characterized as a strong predictor of indoor particulate matter [35–37] and its presence may have obscured the associations between other sources and indoor particulate matter. All home owners provided written informed consent to participate and the home inspection response rate was 78% [34].

### 2.2. Home Inspections and Measurements

All 60 home inspections were carried out by two research assistants. In addition to dust collection and air pollution monitoring, a visual inspection and a home inspection questionnaire (HI questionnaire) were completed for each home. All families were asked not to vacuum for at least four days prior to dust collection.

Dust samples were collected using a Shop-Vac QAM70 vacuum with X-Cell 100 filters and samples were taken from the home’s living space where the child spent most of his/her time when not in the bedroom. An area of 2 m^2^ for carpeted floors and 4 m^2^ for hardwood floors was vacuumed for 3 minutes. Collected samples were analyzed at Paracel Laboratories in Ottawa (Ontario, Canada). After being weighed and sieved to exclude particles greater than 300 μm in size, the dust was analyzed for dust mite, cat and cockroach allergen content using immunoassays employing monoclonal antibodies for Der p 1, Der f 1, Fel d 1, and Bla g 1, respectively [38]. Detection limits were 10 ng/g of dust for Der p 1 and Der f 1, 3.12 ng/g for Fel d 1, and 0.04 U/g for Bla g 1. Mouse allergen concentration was determined using an enzyme-linked immunosorbent assay (ELISA) with purified rabbit polyclonal IgG antibodies specific for mouse urinary protein allergen, Mus m 1. The limit of detection for Mus m 1 was 0.001 μg/g of dust. Bacterial endotoxin was measured by washing with pyrogen free water and submitting the water extracts to Limulus Amoebocyte Lysate (LAL) assay using a chromogenic test kit in a kinetic assay on an MR 5,000 microplate reader [39]. The detection limit for endotoxin was 0.625 ng per gram of dust. The fungal component, (1→3)-β-d-glucan, was measured via the “factor G” LAL-based analytical method [40]. Dust samples were also analyzed specifically for the *Alternaria alternata* fungus, using an ELISA with monoclonal antibodies for Alt a 1. The limit of detection was 62.5 ng/g for glucan and 0.032 μg/g for Alt a 1. There is a lack of consensus as to whether allergen concentrations should be expressed by unit weight of dust or by unit area sampled [41,42]. In order to compare current results to reported values in the literature and to previously reported notional thresholds of sensitization, all results are expressed as both concentrations per gram of dust (μg/g) and per area (μg/m^2^).

Air pollution monitoring was completed over a 6-day period and all measurements were taken as averages over this time period. Air exchange rates within the home were measured using perfluorocarbon emitters (PFT), which were placed in the four corners of the main floor, and a capillary adsorption tube (CAT) detector, which was located in a central location on the main floor [43]. Sampling of indoor and outdoor particulate matter (PM_2.5_) and nitrogen dioxide (NO_2_) concentrations were conducted using an R&P Chempass Multi-pollutant sampler (R&P/Thermo, Waltham, MA, USA) that housed a passive Ogawa badge (Ogawa & Co, FL, USA) [44]. Indoor and outdoor continuous PM_2.5_ was also measured over the 6 days using a DustTrak (Model 8520, TSI, St. Paul, MN, USA).

### 2.3. Data Analysis

Basic bivariate statistics were calculated on all variables using Statistical Analysis Software (SAS) v9.1 (SAS Institute Inc, Cary, NC, USA). Measures of allergens were positively-skewed and were transformed by natural logarithm for data analysis; a square root transformation was used for glucan in μg/g. Pollutants were analyzed as continuous variables. Dust mite and cat allergen were also dichotomized according to previously reported notional thresholds of sensitization (Der p 1 or Der f 1 > 2 μg/g, Fel d 1 > 1 μg/g) and of asthma morbidity (Der p 1 or Der f 1 > 10 μg/g, Fel d 1 > 8 μg/g) [42,45–47]. All data analyses were run using predictors chosen *a priori* based on existing evidence in the literature. Indoor relative humidity (RH) was analyzed as the percentage of time RH was over 50% in the home; this variable was also dichotomized into homes with mean indoor RH above or below 50%. This was done based on current recommendations for dust mite allergen reduction [24] as well as previous work which showed that maintaining a mean daily RH below 50% effectively restricts dust mite growth and allergen production [48]. A linear regression was performed between dust mite levels and relative humidity. To determine whether gas appliances predicted increased indoor NO_2_ levels while adjusting for outdoor NO_2_ and air exchange rate, a multiple regression was performed. Student’s t-tests were employed to compare dichotomous T-CHEQ and HI questionnaire responses with continuous pollutant concentrations. Fisher’s exact tests were employed to assess the relationship between dichotomized pollutant measures and questionnaire responses.

## 3. Results

A summary of respondent and home characteristics as reported in the T-CHEQ and the home inspection (HI) questionnaire is found in Table 1. Of note, cockroaches were reported in only one home; however, Bla g 1 was below the detection limit of 0.04 U/g in all homes except one, where there was an insufficient amount of sampled dust to perform the analysis. Air monitoring measurements are shown in Table 2 and the distribution of allergen levels as measured in dust are shown in Table 3.

### 3.1. Indoor NO_2_

**Figure 1.**
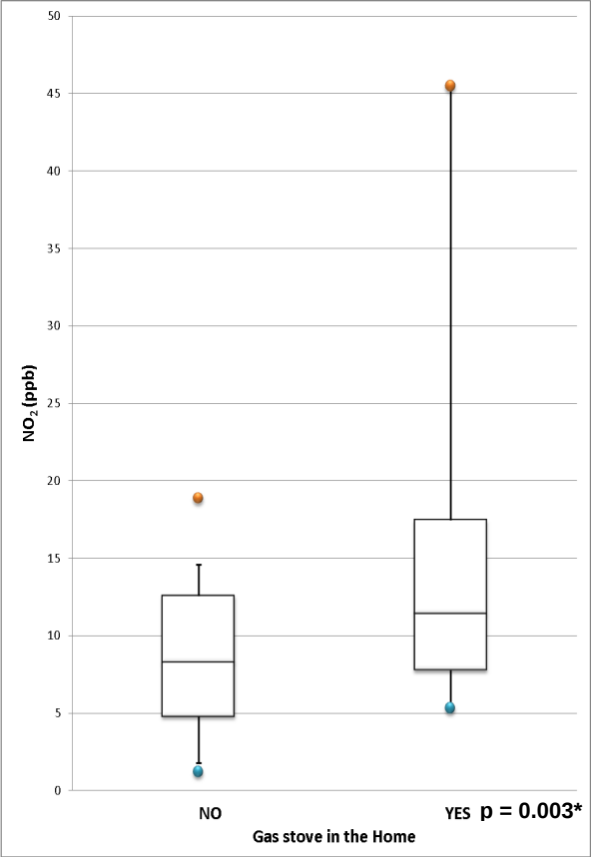
Distribution of NO_2_ (ppb) comparing homes with and without gas stoves. *Two-sample t-test comparing Ln-transformed population means.

Indoor NO_2_ concentrations in all homes were below Health Canada’s acceptable long-term exposure range of ≤50 ppb for indoor air [49]. In homes where gas was the main cooking fuel, there were two homes with particularly high NO_2_ levels (Figure 1).

The maximum value (NO_2_ = 45.5 ppb) was obtained at a home in which the gas stove was not vented. The other outlier (NO_2_ = 40.2 ppb) occurred in a home where there was a gas stove as well as a gas dryer, both of which were vented. A gas stove was designated as “vented” during the home inspection if the range hood above the stove vented directly to the outside. Mean indoor NO_2_ was significantly higher (*p* = 0.003) in homes that reported the main cooking fuel as gas in the T-CHEQ (14.98 ppb) than in homes that reported electricity as the main cooking fuel (8.31 ppb). Report of gas appliances in the HI questionnaire included stove, dryer, fireplace, water heater, and water boiler. In bivariate analyses, only gas stove was a significant predictor of indoor NO_2_. In a multiple regression that accounted for ventilation and outdoor NO_2_ levels, gas stove (*p* = 0.01) remained a significant predictor of indoor NO_2_ (R^2^ = 0.35).

### 3.2. Indoor PM_2.5_

Indoor PM_2.5_ concentrations for all homes were below Health Canada’s acceptable long-term exposure range of ≤40 μg/m^3^ for indoor air [49]. PM_2.5_ levels could not be obtained for one home due to problems with the sampling equipment. No significant predictors of indoor PM_2.5_ were found. In particular, HI questionnaire report of vacuuming frequency in the living room and interior wood storage in the home did not predict indoor PM_2.5_. Measured outdoor PM_2.5_ and air change rates also did not exhibit a significant relationship with indoor PM_2.5_.

### 3.3. Cat Allergens

**Figure 2.**
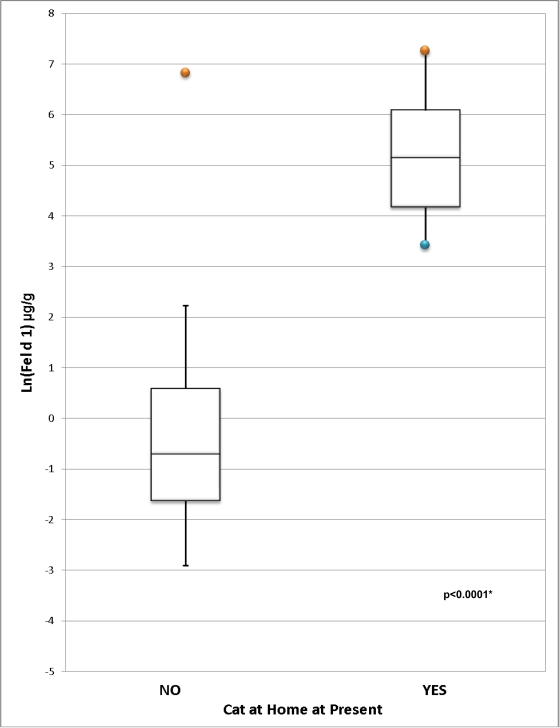

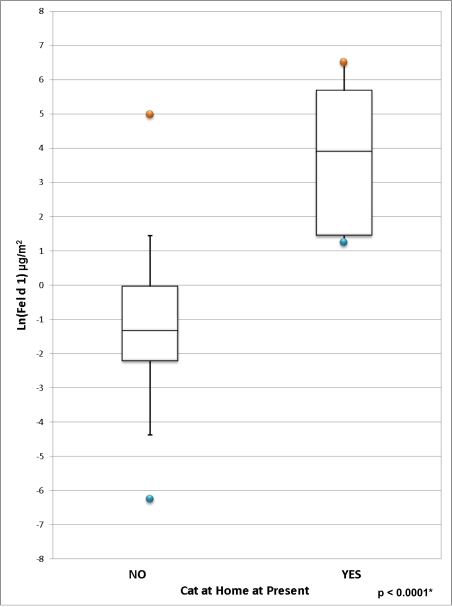
**(a)** Distribution of Ln-transformed Fel d 1 concentration (μg/g) in homes with and without cats. **(b)** Distribution of Ln-transformed Fel d 1 load (μg/m^2^) in homes with and without cats. *Two-sample t-test comparing Ln-transformed population means. (a) * Two-sample t-test comparing Ln-transformed population means. (b)

Detectable levels of Fel d 1 were found in all homes except one, where there was not enough dust to perform this analysis. The distribution of Fel d 1 in homes with and without cats is displayed in Figure 2. Mean Fel d 1 levels were significantly higher (p < 0.0001) in homes with cats (187.82 μg/m^2^, 450.58 μg/g) than in homes without cats (3.63 μg/m^2^, 22.28 μg/g). All homes with a cat had Fel d 1 levels > 8 μg/g, the asthma symptom threshold, whereas only 7.8% of homes without a cat had Fel d 1 levels > 8 μg/g (p < 0.0001, κ = 0.78). By contrast, all 9 homes with a cat had Fel d 1 > 1 μg/g, the notional sensitization threshold; however, 47.1% of homes without a cat also had Fel d 1 > 1 μg/g (p = 0.003, κ = 0.25).

### 3.4. Dust Mite Allergen

Der f 1 was the more prevalent dust mite allergen in the 60 homes sampled. Detectable levels of Der f 1 were found in all homes; one home did not have enough sample available to perform this analysis. By contrast, Der p 1 was below the detection limit of 10 ng/g in 27.6% of homes, and below the notional sensitization threshold of 2 μg/g in 91.6% of homes.

No significant predictors of Der p 1 were found. Results of bivariate analyses of predictors of Der f 1 are displayed in Table 4. Mean Der f 1 levels were significantly higher in homes built before 1990 compared to those built after 1990 (47.61 μg/g *versus* 10.96 μg/g, *p* = 0.043; 17.54 μg/m^2^, *versus* 0.2 μg/m^2^, *p* = 0.012)

**Figure 3.**
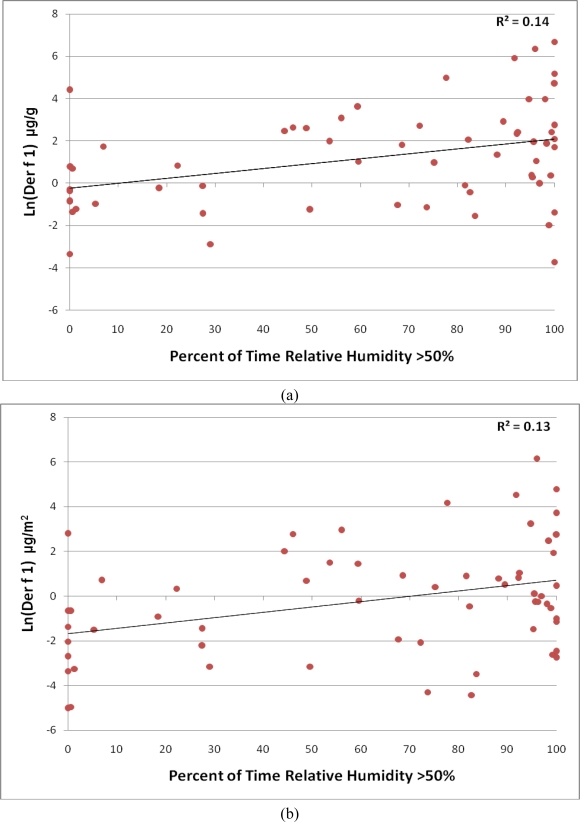
**(a)** Ln-transformed Der f 1 (μg/g) concentration by percent of time relative humidity exceeds 50%. **(b)** Ln-Transformed Der f 1 load (μg/m^2^) by percent of time relative humidity exceeds 50%.

**Figure 4.**
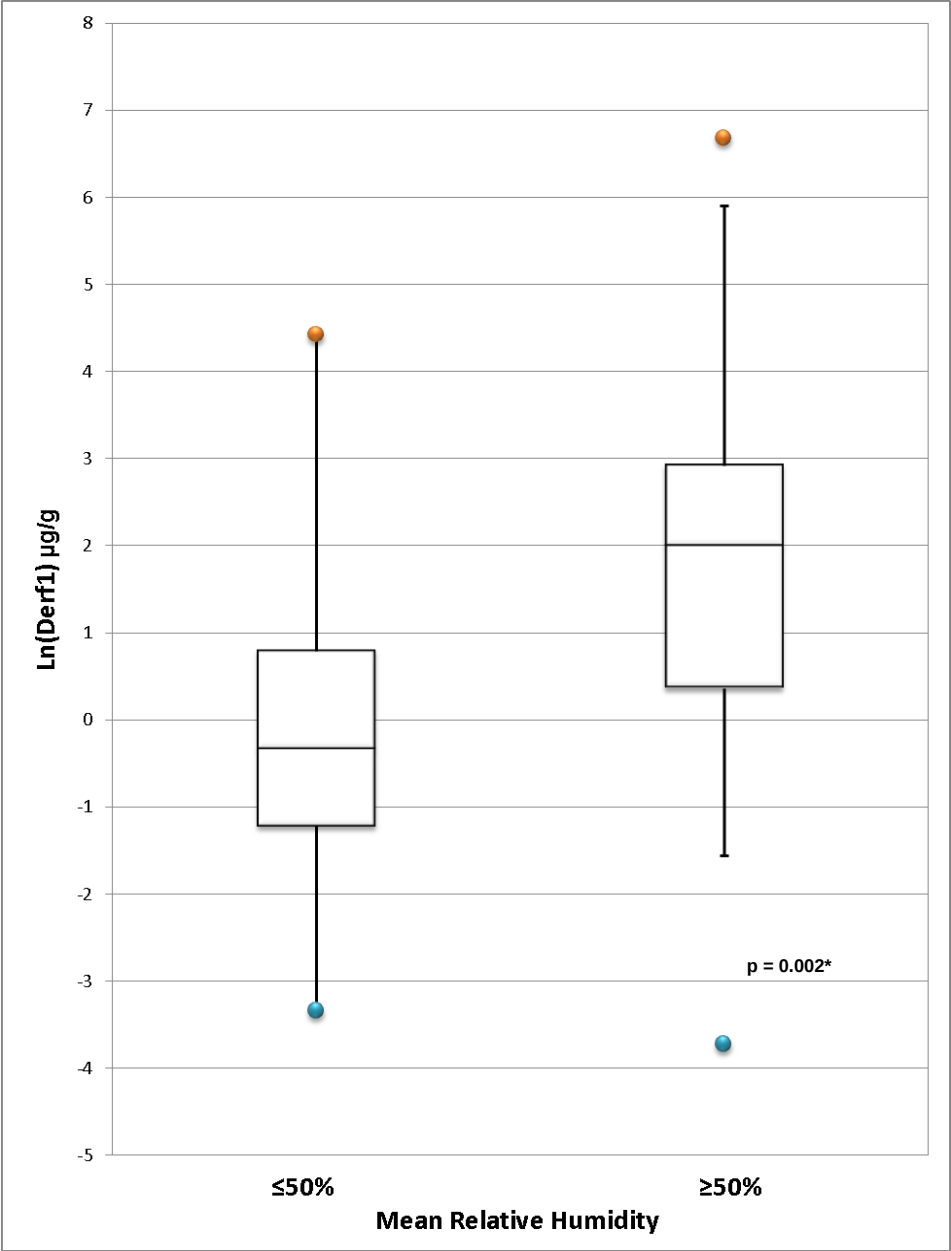

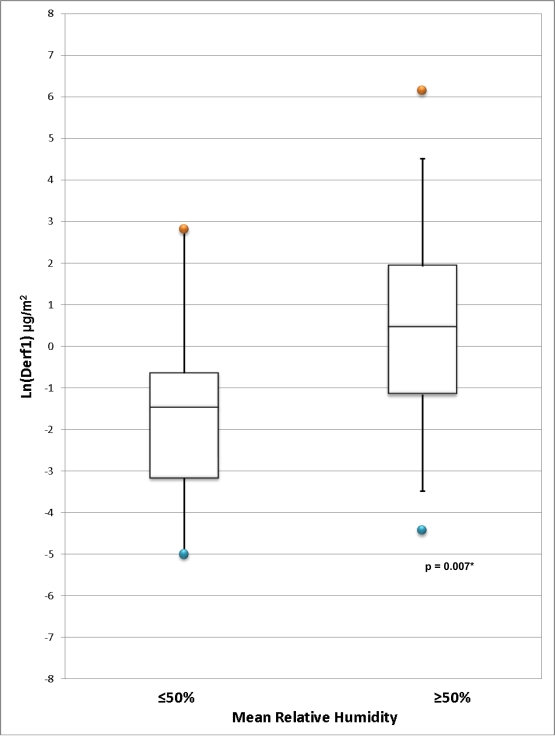
**(a)** Distribution of Ln-transformed Der f 1 concentration (μg/g) in homes comparing mean relative humidity. **(b)** Distribution of Ln-Transformed Der f 1 load (μg/m^2^) in homes comparing mean relative humidity. *Two-sample t-test comparing Ln-transformed population means. (a) *Two-sample t-test comparing Ln-transformed population means. (b)

A sampling surface of carpet, compared to hardwood, predicted dust weight (1.13 g *versus* 0.30 g; *p* < 0.001), but not Der f 1 levels expressed as μg/g (*p* > 0.6); carpet sampling surface approached significance as a predictor of Der f 1 levels expressed as μg/m^2^ (*p* = 0.06). Homes that reported using central air conditioning for less than 30 days per year had higher mean Der f 1 levels expressed as μg/m^2^ compared to homes that reported using central air conditioning for at least 30 days per year (29.50 μg/m^2^ *versus* 5.20 μg/m^2^; *p* = 0.009). A significant, moderate correlation was found between Der f 1 levels in μg/g and the percentage of time in which indoor relative humidity was above 50% (*p* = 0.002, r = 0.39; Figure 3A). This relationship was also significant for Der f 1 levels in μg/m^2^ (*p* = 0.005, r = 0.36; Figure 3B). The distribution of Der f 1 in homes with mean RH above and below 50% is displayed in Figure 4. Der f 1 levels were significantly higher in homes with mean RH over 50% (61.30 μg/g *versus* 6.24 μg/g, *p* = 0.002; 22.39 μg/m^2^ *versus* 1.68 μg/m^2^, *p* = 0.007).

### 3.5. Mouse Allergens

**Figure 5.**
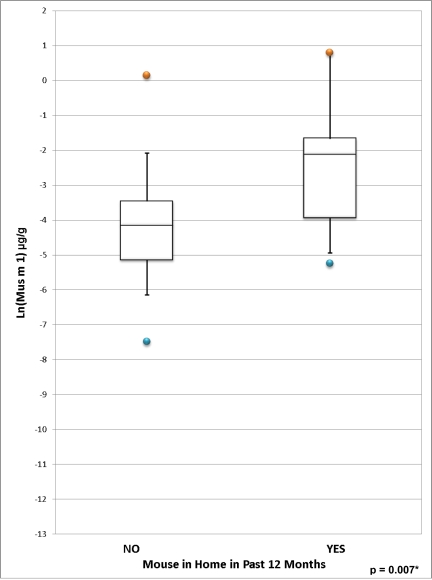

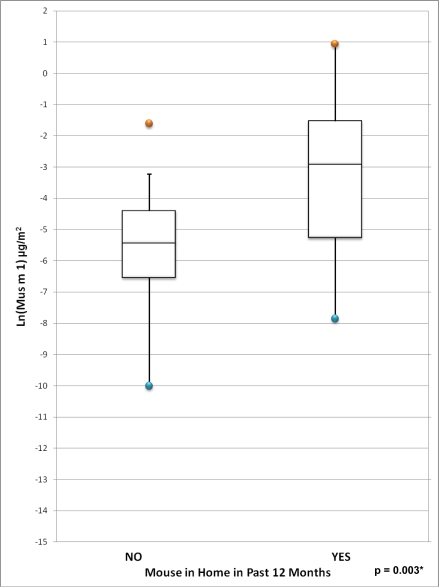
**(a)** Distribution of Ln-transformed Mus m 1 concentration (μg/g) in homes with and without mice in the past 12 months. **(b)** Distribution of Ln-transformed Mus m 1 Load (μg/m^2^) in homes with and without mice in the past 12 months. *Two-sample t-test comparing Ln-transformed population means. (a) *Two-sample t-test comparing Ln-transformed population means. (b)

Detectable levels of Mus m 1 were found in all homes except eight, where there was insufficient dust to perform the analysis. Figure 5 displays the distribution of Mus m 1 in homes that did *versus* did not report mice as pests in the past 12 months. Mean Mus m 1 was significantly higher in homes that reported mice as pests (0.36 μg/g *versus* 0.06 μg/g, *p* = 0.007; 0.36 μg/m^2^ *versus* 0.02 μg/m^2^, *p* = 0.003). Reported age of the home in the HI questionnaire was not predictive of Mus m 1. No significant association was found between the reported presence of a cat in the home and Mus m 1 levels.

### 3.6. Endotoxin

Detectable levels of endotoxin were found in all 60 homes. The presence of a cat, a dog, or any pet in the home, as reported in the T-CHEQ, did not predict levels of endotoxin in μg/g or μg/m^2^.

### 3.7. Mould

**Figure 6.**
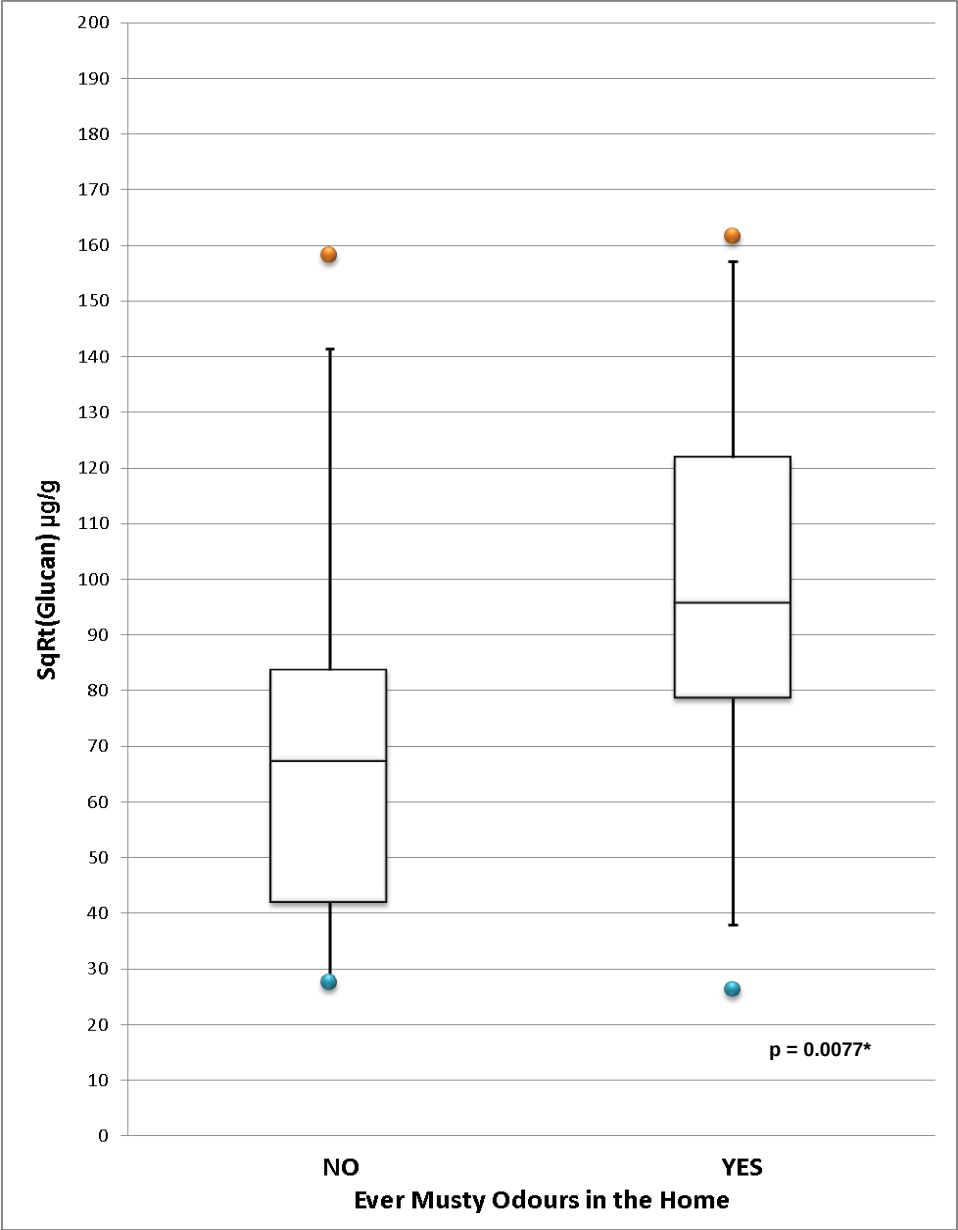

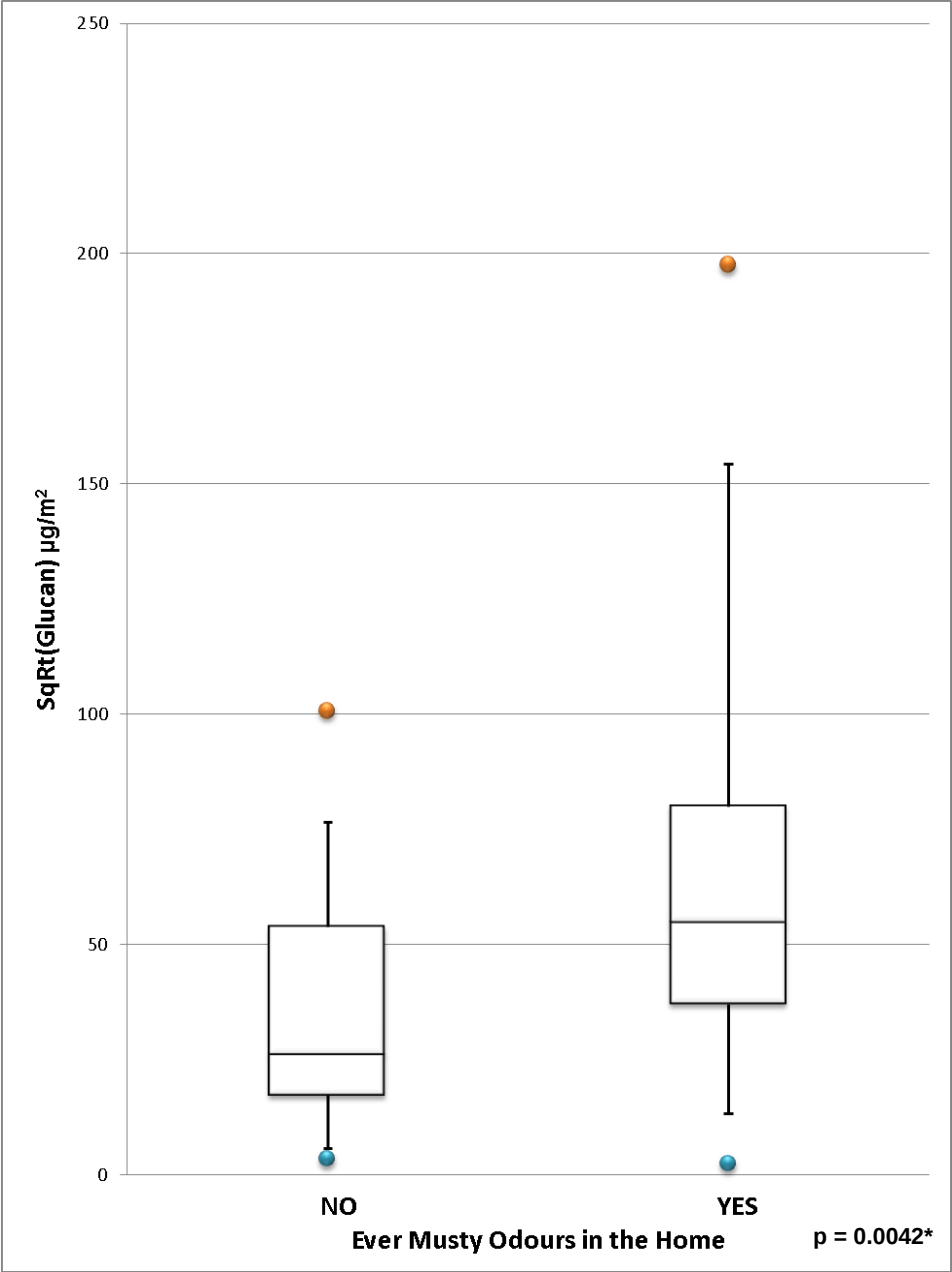
**(a)** Distribution of Glucan Concentration (μg/g) in Homes Reporting and Not Reporting Musty Odours. **(b)** Distribution of Glucan Load (μg/m^2^) in Homes Reporting and Not Reporting Musty Odours. *Two-sample t-test comparing square root transformed population means. (a) *Two-sample t-test comparing square root transformed population means. (b)

Alt a 1 was below the detection limit of 32 ng/g in all homes, with the exception of 7 houses, where there was insufficient dust to perform the analysis. However, detectable levels of glucan were present in all homes except 4, where there was insufficient dust to perform this analysis. Figure 6 displays the distribution of glucan in homes according to whether a musty odour was reported. Of note, 34 homes reported experiencing musty odours in the home in the HI questionnaire, but in the T-CHEQ, only 1 reported damp spots and 3 reported mould. Report of musty odours in the HI questionnaire was a significant predictor of glucan levels (10,554.37 μg/g *versus* 6,308.58 μg/g, *p* = 0.0077; 6,173.62 μg/m^2^ *versus* 1,999.73 μg/m^2^, *p* = 0.042).

## 4. Discussion

Self-reported age of the home, presence of a gas stove, a cat, mice as pests, and musty odours from questionnaire data predicted objectively measured levels of pollutants in the home that may be associated with adverse health outcomes. No other self-reported home characteristics were predictive of potentially harmful pollutants. This has relevance both for epidemiological studies that assess the effects of indoor air pollutants on adverse health outcomes, as well as for clinical practice, where simple questions may be used to assess harmful exposures at home. Although neither a carpet sampling surface nor the presence of an air conditioner predicted increased levels of dust mite allergen, relative humidity above 50% predicted higher dust mite levels, supporting current guideline recommendations of reducing relative humidity as part of allergen exposure reduction [50,51].

### 4.1. Strengths and Limitations

Few studies have collected both dust and air quality measurements in the same homes. Our analysis of indoor pollutants also includes Mus m 1 which have not been well-characterized previously, especially in Canada.

One of the main strengths of this study lies in its population-based sampling approach. However, because all sampled homes were owned and not rented, the results may not reflect conditions in types of dwellings such as multi-unit apartments that are more likely to be rented than owned. Restricting the sample to owned dwellings also skewed it to households with higher incomes. In the 2001 Canadian Census, data for the Toronto census metropolitan area restricted to households with at least one child aged 5 to 17 years revealed that 43.5% of this population were in the highest income adequacy group [52]. 37% of the overall T-CHEQ population (*n* = 5619) was in the highest income category, while 71% of the home inspection subsample (*n* = 60) was in the highest income category. While this indicates that our results may not be readily generalizable to the wider population, it is interesting to note that we found detectable and even high levels of certain pollutants, such as cat and dust mite allergens in a mostly high-income group of homes. On the other hand, for pollutants such as NO_2_ and mouse allergen, even when significant associations existed between specific home characteristics and increased pollutants, overall concentrations tended to be low compared to reports from other studies. As a result, in our study population, although some self-reported characteristics via questionnaire were found to be predictive of objective measures of the corresponding pollutant, they were not necessarily indicative of pollutant levels that may lead to adverse health outcomes.

### 4.2. Indoor NO_2_

Questionnaire report of a gas stove, in conjunction with air change rates and outdoor NO_2_ contributions accounted for about one-third of the variance (R^2^ = 0.35) in indoor NO_2_ levels. In a previous study in Quebec City, gas heating systems, gas stoves, and air change rates were identified as significant predictors of indoor NO_2_ concentrations, explaining 48% of the variance in a multiple regression model [53]. Gas furnace was not included as a predictor in our model for indoor NO_2_ because measurements were conducted in the summer and fall, when furnaces were not in operation.

In our study, the measured indoor NO_2_ concentrations were relatively low, even in homes with gas appliances (mean NO_2_ = 12.1 ppb). Similar levels were also observed in the Stockholm BAMSE birth cohort in homes with gas stoves (mean NO_2_ = 12.0 ppb) [54]. By contrast, recent U.S. studies have reported markedly higher indoor NO_2_ concentrations in homes with gas stoves and have found increased NO_2_ levels to be associated with asthma morbidity [55,56]. However, these study populations were primarily composed of inner city residents and lower income groups, and many homes had gas stoves that were not vented [56]. Consequently, in our study, although the presence of a gas stove in the home was predictive of increased NO_2_, the presence of these gas appliances may not necessarily reflect exposures that would lead to adverse health outcomes.

### 4.3. Indoor PM_2.5_

No self-reported home characteristics were found to predict indoor PM_2.5_ in either the T-CHEQ or the HI questionnaire. Because all home inspections were conducted in the summer or fall, the effect of operating wood-burning fireplaces could not be effectively examined. Outdoor PM_2.5_ and air change rates did not predict indoor PM_2.5_ concentrations, although other studies have found both indoor sources such as cooking and cleaning, and outdoor sources to contribute to PM_2.5_ levels inside the home; air change rates have also been found to modulate the extent to which indoor PM_2.5_ levels reflect outdoor or indoor sources [57,58]. Human activities in the home, such as walking or vacuuming were not assessed in detail via questionnaire; however, these activities have been shown to contribute to indoor particulate matter via the resuspension of house dust [59]. More recently, increased early life exposure to traffic-related air pollutants have been found to increase the risk of asthma diagnosis [60] and self-report of truck traffic on the street of residence has also been positively associated with increased report of asthma symptoms [61]. Future research would be of interest to determine whether a questionnaire report of traffic in the vicinity of the home is a good predictor of measured concentrations of particulate matter and other traffic-related pollutants both inside and outside the home.

### 4.4. Cat Allergen

The indication of a cat in the home via questionnaire was found to predict increased cat allergen as a continuous variable expressed in μg/g and μg/m^2^, as well as cat allergen levels above 1 μg/g and above 8 μg/g. This is consistent with a previous study of homes in the Boston area, in which report of one or more cats currently in the home was found to be the best predictor for Fel d 1 levels ≥ 1 and ≥8 μg/g of dust [26]. The presence of cat allergen above the notional sensitization threshold in 47.1% of homes without cats, and above the asthma symptom threshold in 7.8% of homes without cats, suggest that cat allergen burden within the home may not be solely attributable to a cat currently in the home. The US National Survey of Lead and Allergens in Housing (NSLAH) also found that, for Fel d 1, 55.7% of homes without an indoor cat were above the notional sensitization threshold and 15.7% of homes without an indoor cat were above the asthma symptom threshold [45]. Previous work has shown that clothing and automobiles can be vehicles of pet allergen dispersal [62] and it is therefore likely that the tracking in of cat allergen from outdoors contributes to cat allergen concentrations in the home. Therefore, while report of a cat in the T-CHEQ is a good predictor of high cat allergen, report of no cat in the home is a poor indication of the absence of cat allergen. With respect to health implications, although exposure to cat allergen can exacerbate asthma symptoms in sensitized individuals, and may also lead to a higher risk of developing cat sensitization in children, current evidence remains inconclusive as to whether an association exists between cat exposure and the development of asthma itself [63,64].

### 4.5. Dust Mite Allergen

Having an older home built prior to 1990 was a significant predictor of increased Der f 1 levels. Older homes were also a significant predictor of dust mite allergen in the NSLAH [65] and in a recent pooled analysis of nine U.S. asthma studies [29]. The T-CHEQ question on flooring pertained only to the child’s bedroom, whereas dust sampling was conducted in the living space where the child spent most of his/her time when not in the bedroom. Neither report of carpets in the child’s bedroom nor a sampling surface of carpet were significant predictors of dust mite allergen concentrations, although previous studies have found them predictive of increased Der p 1 and Der f 1 levels [5,26]. The low levels of Der p 1 that were found in this study are similar to what has been previously reported for Toronto in the Child Asthma Management Program (CAMP), where over 90% of homes had Der p 1 below the notional sensitization threshold and Der f 1 was the predominant dust mite allergen [66]. Although report of an air conditioner in the home was not predictive of dust mite allergen, the percentage of time that measured indoor relative humidity was above 50% was significantly associated with Der f 1 levels. Our results are consistent with previous findings that a mean RH below 50% effectively restricts dust mite growth [48], and our findings support current guideline recommendations to reduce dust mite allergen by maintaining RH below 50% [24,48].

### 4.6. Cockroach Allergen

Only one home reported the presence of cockroaches and Bla g 1 levels were below the detection limit of 0.04 U/g in all homes. The absence of cockroach allergen may be attributable to the fact that all homes in this study were owned, single-family dwellings; in a study of homes in the Boston area, the odds of recovering detectable levels of cockroach allergen (Bla g 1 or Bla g 2 ≥ 0.025 U/g) have been found to be lower for houses and duplexes compared to apartments [26]. Furthermore, in the CAMP study, cockroach allergen was undetectable (Bla g 1 < 0.4 U/g) in 97.5% of Toronto homes (*n* = 118). The relatively small sample size of 60 homes in this study may have limited our ability to detect cockroach allergen.

### 4.7. Mouse Allergen

Report of mice as pests in the past 12 months in the home inspection questionnaire was found to predict increased levels of mouse allergen expressed as both μg/g and μg/m^2^. This is consistent with the results of a Baltimore inner-city study, where mouse infestation predicted detectable mouse allergen [67]. Reports of rodents were also associated with increased mouse allergen in both the NSLAH and a pooled analysis of nine U.S. asthma studies [29,68]. The presence of detectable mouse allergen in all homes with sufficient sample to perform this analysis suggests that this pollutant is quite prevalent in Toronto homes, despite the large proportion of high-income households in this study population. However, indoor levels of Mus m 1 in this study, even in homes that reported mice as pests, were markedly lower than values reported for US homes [22,68,69]. Only three homes had Mus m 1 concentrations above 0.25 μg/g, of which two homes had Mus m 1 concentrations above 0.5 μg/g—concentrations that have been previously associated with allergic sensitization and allergic symptoms [22]. Because sensitization and asthma symptom thresholds have not been firmly established for this allergen, it is unclear whether the low, widespread levels of mouse allergen in this study, and even the relatively increased levels in homes reporting mice as pests, have significant adverse health effects.

### 4.8. Endotoxin

Report of a cat, a dog, or any pet in the T-CHEQ did not predict increased levels of endotoxin in the home. Current evidence in the literature is not conclusive with respect to the predictors of endotoxin levels. In a study of 405 German homes, the presence of a cat, the presence of a dog, and poor hygienic conditions in the home were associated with high endotoxin concentrations in settled dust [70]. The Cincinnati Childhood Asthma and Air Pollution study of 532 homes reported that while homes with dogs alone and cats alone exhibited higher endotoxin loads compared to homes without pets, this relationship was only statistically significant for homes with dogs [30]. In the AIRALLERG study of homes of 1,065 German, Dutch and Swedish pre-school children, although there was an association between high endotoxin levels in floor dust and the presence of a cat, a dog, and other pets in the home, not all of these associations were statistically significant for all three countries [31]. The AIRALLERG study results also showed that, in general, the questionnaire variables on housing characteristics accounted for a low proportion of variance in endotoxin concentrations and did not accurately predict endotoxin concentrations in house dust [31].

### 4.9. Mould

Report of musty odours in the HI questionnaire was a significant predictor of glucan levels in the home. In the T-CHEQ, however, low reporting of dampness or mould in the home, even in the presence of detectable glucan levels, suggests that these questions may not be accurate predictors of actual fungi levels in the home. Questionnaire report of mould, musty odours, or moisture-related problems have been previously associated with levels of fungi in dust in the NSLAH [28] and in a study of 403 homes in Wallaceburg, Ontario [71]. The discrepancy between parental T-CHEQ responses and actual mould in the home may indicate that, by only ascertaining the presence or absence of mould and dampness, the T-CHEQ questions are not sufficiently detailed to capture the true conditions of the home environment. Alternatively, the discrepancy may be due to the inability of residents to detect mould on hidden surfaces or within walls. It may also reflect the potential unwillingness of parents to report the presence of such factors that are known to be detrimental to health, particularly within the context of a questionnaire related to their child’s health. The latter may have been the case in the home with the highest level of glucan, where mould was not reported in the T-CHEQ, but was identified by the resident to research assistants during the home inspection.

Our findings that *A alternata* allergen was below the detection limit of 0.032 μg/g in all homes contrast with the results of the NSLAH, which found *Alternaria* antigens in 95% to 99% of dust samples from US homes [28]. It is possible that geographic and climatic differences may explain the low prevalence of *A alternata* as an indoor fungus in Toronto homes. However, Schmechel *et al.* recently reported that the polyclonal antibodies used to detect *A alternata* in the NSLAH cross-reacted broadly with other fungi and were not therefore *Alternaria*-specific [72]. A species-specific monoclonal antibody was used to detect the Alt a 1 allergen in this study and it is therefore possible that the indoor prevalence of the *A alternata* fungus is indeed quite low in our study population.

## 5. Conclusions

Questionnaire reports of a cat, of a gas stove, of mice as pests, of musty odours, and of the home’s age predicted objectively measured concentrations of pollutants in the home at levels that may be associated with adverse health outcomes. No other questions pertaining to home characteristics were predictive of potentially harmful pollutant exposures. This highlights the importance of using valid questions in large epidemiological surveys that assess the effects of indoor air pollutants on adverse health outcomes. These results are also relevant for clinical practice, and suggest that single questions, such as whether there are carpets in the house, may not be sufficient to provide an accurate assessment of harmful exposures at home.

## Figures and Tables

**Table 1.** Participant and Dwelling Characteristics.

**VARIABLE**	T-CHEQ PHASE 3 (n = 60)*		T-CHEQ PHASE 1 (N = 5559)*	

	***Frequency (%)***	**95%*Confidence Limits***	***Frequency (%)***	**95%*Confidence Limits***
***PARTICIPANT CHARACTERISTICS***				
**Sex**				
Male	28 (46.7)	(33.7, 59.7)	2,744 (50.2)	(48.9, 51.5)
**Household Income Adequacy**				
Lowest	0 (0)	-	935 (18.0)	(17.0, 19.1) ‡
Lower Middle Income	4 (6.8)	(0.2, 13.4)	1,156 (22.3)	(21.2, 23.4) ‡
Upper Middle Income	13 (22.0)	(11.1, 32.9)	1,188 (22.9)	(21.8, 24.1)
Highest Income	42 (71.2)	(59.3, 83.1)	1,904 (36.7)	(35.4, 38.0) ‡
**Lifetime Asthma**	11 (18.3)	(8.3, 28.4)	836 (15.5)	(14.5, 16.4)
***DWELLING CHARACTERISTICS***				
**Type Of Dwelling**				
Single Detached House	50 (83.3)	(73.6, 93.0)	2,359 (44.6)	(43.3, 46.0) ‡
Double (Semi Detached)	7 (11.7)	(3.3, 20.0)	746 (14.1)	(13.2, 15.1)
Row Or Terrace House	3 (5.0)	(0.0, 10.7)	282 (5.3)	(4.7, 5.9)
Duplex/triplex/low rise apt (<=5 stories)	EXCLUDED		446 (8.4)	(7.7, 9.2)
High rise (>5 stories)	EXCLUDED		1,368 (25.9)	(24.7, 27.1)
Institution, hotel; rooming/lodging	EXCLUDED		84 (1.6)	(1.3, 1.9)
house/camp, mobile home, other						
**Year Dwelling Built**				
1990 Or Later	5 (8.3)	(1.1, 15.5)	NA	NA
1969 To 1989	9 (15.0)	(5.7, 24.3)	NA	NA
1949 To 1969	16 (26.7)	(15.1, 38.2)	NA	NA
Before 1949	30 (50.0)	(37.0, 63.0)	NA	NA
**Cooking Fuel Used At Present**†				
Gas	19 (31.7)	(19.5, 43.8)	1,066 (19.6)	(18.5, 20.7)
Electric	41 (68.3)	(56.2, 80.5)	4,374 (80.4)	(79.3, 81.5)
**Cat In Home At Present**†	9 (15.0)	(5.7, 24.3)	751 (13.5)	(12.6, 14.4)
**Any Pets At Home At Present**†	38 (63.3)	(50.8, 75.9)	1,495 (29.4)	(28.1, 30.6) ‡
**Carpets in Child’s Bedroom**	46 (76.7)	(65.4, 87.7)	2,358 (42.4)	(41.1, 43.7) ‡
**Air Conditioning**	58 (96.7)	(92.0, 100.0)	4,074 (73.3)	(72.1, 74.5) ‡
**Roaches At Present**†	1 (1.7)	(0, 5.0)	546 (9.8)	(9.0, 10.6) ‡
**Mice At Present**	10 (16.7)	(7.0, 26.4)	5,013 (90.2)	(9.0, 10.6)
**Damp Spots In House At Present†**	7 (11.7)	(3.3, 20.0)	384 (6.9)	(6.2, 7.6)
**Mould In House At Present**	28 (46.7)	(33.7, 59.7)	336 (6.0)	(5.4, 6.7)

* T-CHEQ sample size (excludes missing data). T-CHEQ sample has previously been shown to be representative of the population of grades 1 and 2 school children living in Toronto in 2006 [34].

** Income adequacy: a derived variable defined by Statistics Canada as (income, persons in household) Lowest income: <$15,000, 1–2 or <$20,000, 3–4 or <$30,000, 5+; Lower middle income: $15,000 to $29,999, 1–2, or $20,000 to $39,999, 3–4, or $30,000 to $59,999, 5+; Upper middle income: $30,000 to $59,999, 1–2, or $40,000 to $79,999, 3–4, or $60,000+, 1–2, or $80,000+, 3+ ; Highest income: $60,000+, 1 or 2, or $80,000+, 3+.

† “Present” refers to 2006 in the case of T-CHEQ Phase 1, and 2007–08 in the case of Phase 3 .

‡ Statiscically Significant difference based on non-overlapping 95% confidence intervals NA: Not Available

**Table 2.** Levels if NO_2_, PM_2.5_ and air monitoring measurements in homes.

	
	**Mean (SD)**	**Median**
Indoor temperature (°C)	22.5 (2.1)	22.6
Indoor relative humidity (%)	51.7 (7.4)	50.7
Ventilation (air changes/hour)	0.36 (0.34)	0.30
Indoor NO_2_ (ppb)	10.0 (7.5)	8.6
Outdoor NO_2_ (ppb)	15.6 (4.8)	14.9
Indoor PM_2.5_ (μg/m^3^)	9.2 (5.6)	7.8
Outdoor PM_2.5_ (μg/m^3^)	9.6 (3.7)	9.0

**Table 3.** Distribution and levels of pollutants in dust samples.

	**N**	**%**	**Mean (SD) [μg/g]**	**Median [μg/g]**	**Mean (SD) [μg/m^2^]**	**Median [μg/m^2^]**
**Fel d 1**			87.62 (268.52)	1.42	31.73 (118.08)	0.49
Insufficient dust to perform analysis	1	1.67				
≤1 μg/g	26	43.33				
>1 μg/g to 8 μg/g	20	33.33				
>8 μg/g	13	21.67				
**Der p 1**			0.60 (1.90)	0.04	0.29 (0.91)	0.02
Insufficient dust to perform analysis	2	3.33				
Below detection limit (<0.01 μg/g)	16	26.67				
10 ng/g to ≤2 μg/g	39	65.00				
>2 μg/g to 10 μg/g	2	3.33				
>10 μg/g	1	1.67				
**Der f 1**			44.50 (135.05)	2.87	16.07 (63.79)	0.77
Insufficient dust to perform analysis	1	1.67				
≤2 μg/g	25	41.67				
>2 μg/g to 10 μg/g	14	23.33				
>10 μg/g	20	33.33				
**Bla g 1**			NA	NA	NA	NA
Insufficient dust to perform analysis	1	1.7				
Below detection limit (<0.04 U/g)	59	98.3				
**Mus m 1**			0.11(0.34)	0.02	0.08 (0.36)	0.01
Insufficient dust to perform analysis	8	13.33				
=<0.001 μg/g	1	1.67				
Above detection limit (>0.001 μg/g)	51	85.00				
**Endotoxin**			9.68 (6.67)	8.93		
Above detection limit (>0.000625 μg/g)	60	100.00				
**(1→3)-β-D-glucan**			8,734.74 (6,985.22)	6,562.32	4,384.81 (6,932.37)	1,939.75
Insufficient dust to perform analysis	4	6.67				
Above detection limit (>0.0625 μg/g)	56	93.33				
**Alt a 1**			NA	NA	NA	NA
Insufficient dust to perform analysis	7	11.7				
Below detection limit (<0.032 μg/g)	53	88.3				

NA: Not available.

**Table 4.** Univariate predictors of dust mite allergen (Der f 1) in the living space of the home where the child spent most of his/her time when not in the bedroom.

	
	**N**	**Der f 1 (μg/g)**		**Der f 1 (μg/m^2^)**	
		**Mean**	**p-value**	**Mean**	**p-value**
**Self-reported variables from the T-CHEQ**					
** Any carpet in the child’s bedroom**			0.95		0.49
Yes	31	45.09		21.41	
No	28	43.86		10.16	
** Air conditioning in the home at present**			0.25		0.16
Yes	51	40.29		15.81	
No	8	71.35		17.73	
** Total number of people in the household**			0.63		0.52
3 or fewer	11	28.26		7.65	
4	27	44.63		9.40	
5	17	63.70		36.04	
6 or more	4	4.36		0.23	
**Report from HI questionnaire**					
** Year home was built**					
Pre-1990	54	47.61	0.04*	17.53	0.01*
1990 or later	5	10.96		0.20	
** Frequency of humidifier use**			0.23		0.56
Never	23	46.07		12.80	
Yes for less than 60 days	9	26.11		19.63	
Yes for at least 60 days	26	98.60		15.91	
** Frequency of central air conditioning use**					
Less than 30 days per year	20	46.36	0.06	29.51	0.01*
At least 30 days per year	29	33.82		5.20	
**Variables from home inspection measurements**					
** Sampling surface**			0.67		0.06
Carpet	47	50.73		19.20	
Hardwood	10	14.02		0.74	
** Calendar season**			0.96		0.80
Summer	32	51.62		20.98	
Fall	27	32.54		7.81	
** Mean indoor relative humidity**			0.002*		0.007*
Mean RH ≤ 50%	18	6.24		1.68	
Mean RH > 50%	14	61.30		22.39	

	**Correlation coefficient (r)**			**Correlation coefficient (r)**	

Percent of time indoor RH > 50%	0.39		0.002*	0.36	0.005*
Mean indoor temperature	−0.01		0.95	−0.04	0.75

* Significant predictor of Der f 1
